# Roles and functions of HIV-1 Tat protein in the CNS: an overview

**DOI:** 10.1186/1743-422X-10-358

**Published:** 2013-12-21

**Authors:** Asen Bagashev, Bassel E Sawaya

**Affiliations:** 1 Molecular Studies of Neurodegenerative Diseases Lab, The Fels Institute for Cancer Research & Molecular Biology, Philadelphia, PA 19140, USA; 2 Department of Anatomy and Cell Biology, Philadelphia, PA 19140, USA; 3 Departments of Neurology, Temple University School of Medicine, Philadelphia, PA 19140, USA

## Abstract

Nearly 50% of HIV-infected individuals suffer from some form of HIV-associated neurocognitive disorders (HAND). HIV-1 Tat (a key HIV *t*rans*a*ctivator of *t*ranscription) protein is one of the first HIV proteins to be expressed after infection occurs and is absolutely required for the initiation of the HIV genome transcription. In addition to its canonical functions, various studies have shown the deleterious role of HIV-1 Tat in the development and progression of HAND. Within the CNS, only specific cell types can support productive viral replication (astrocytes and microglia), however Tat protein can be released form infected cells to affects HIV non-permissive cells such as neurons. Therefore, in this review, we will summarize the functions of HIV-1 Tat proteins in neural cells and its ability to promote HAND.

## Review

### Introduction

The HIV-1 *Tat* (transactivator of transcription) gene codes for a 14-kDa protein and as its name suggests, it is a key activator of HIV-1 transcription. It is one of the first proteins to be expressed after infection occurs. Unlike typical transcription factors that are DNA binding proteins, Tat is a RNA binding protein that recognizes a specific sequence, TAR (*T*rans*a*ctivator *R*esponse Element), from the HIV-1 RNA molecule [1]. Tat is the protein responsible for the recruitment of the host positive transcription elongation factor b (p-TEFb) to the RNA hairpin formed at the 5’-end of nascent viral RNAs (TAR) [2,3]. P-TEFb is a complex composed of cdk9 and cyclin T1 (CycT1) subunits that play a key role in regulating RNA polymerase II dependent transcription. Tat mediated recruitment of p-TEFb drives the phosphorylation of the C-terminal domain (CTD) repeats of RNAP II by cdk9. In its inactive form, p-TEFb binds to the inhibitory 7SK snRNP complex, which can be dissociated by Tat in order to activate cdk9. In neurons, Tat has been linked to progressive neuronal deregulation leading to the development of HIV-Associated Neurocognitive Disorders (HAND) and accelerating brain aging [4]. Although the deregulatory effect of Tat protein in the Central Nervous System (CNS) has been studied extensively, the molecular mechanisms involved remain to be elucidated. In this review we aim to summarize not only the conventional functions of HIV-1 Tat, but its contributing role in the overall complex picture of HAND as well.

### Conventional functions of HIV-1 Tat protein

Tat stimulates HIV-1 gene expression during transcription initiation and elongation. It contains a very strong transcriptional activation domain composed of a Cys-rich region and a hydrophobic core motif, along with an arginine-rich RNA-binding motif (ARM) that specifies the binding of Tat to a base triple in the bulge region of the TAR RNA structure [5]. Binding of purified Tat to TAR-RNA does not require the *cis*-acting sequence within the loop of the TAR structure. The interaction of Tat with a transcriptional co-activator is required for high affinity, loop-specific binding to TAR RNA [6]. The Tat transactivation domain can function independently of the ARM when tethered to the DNA-or RNA- binding domain of a heterologous protein [5]. Although HIV-1 transactivation by Tat in most cell types requires intact TAR sequences, previous reports demonstrated that Tat activates HIV-1 long terminal repeat (LTR)-directed gene expression in several central nervous system-derived astrocytic/glial cell lines in the absence of TAR [7]. Furthermore, genetic experiments have suggested that Tat transactivation of the human immunodeficiency virus type 1 (HIV-1) LTR requires functional upstream enhancer sequences: the kappa B and GC-rich regions. Experiments done with HeLa cell nuclear extracts when using matrices containing chemically synthesized or bacterially expressed HIV-1 Tat, revealed the presence of the Sp1 transcription factor as one of the Tat-binding cellular proteins. Other transcription factors (Oct and NF-κB) also bound to Tat matrices but with less affinity [8].

Tat enhances the processivity of RNA polymerase II complexes that would otherwise terminate transcription prematurely and generate short transcripts, and is thus required to stimulate efficient elongation of viral transcripts [6]. Recently, a new elongation factors were identified, as part of two main protein complexes: TATcom1, which includes the p-TEFb (positive transcription elongation factor b), MLL-fusion partners involved in leukemia (AF9, AFF4, AFF1, ENL, and ELL), and the PAF1 complex [5,9]. This 42-kDa protein was later known as cdk9 which binds to 87-kDa protein, cyclin C-related protein, designated Cyclin T [5]. Neither cdk9 nor cyclin T, cyclin K and Tat activity appear to be cell cycle regulated (this is also true for TFIIH and cdk8 -other factors involved in the phosphorylation elongation phenomenon-) [10]. The addition of Tat to nuclear extracts induces the binding of cdk9-containing TAK/p-TEFb complexes to TAR RNA. Moreover, the nuclear Tat-TAK/p-TEFb complexes did not associate with loop mutant TAR RNA, indicating that the interaction of Tat with TAK/p-TEFb might alter its RNA-binding specificity [5]. While Tat/p-TEFB complexes bind to TAR, cdk9 modifies RNA polymerase II for the efficient copying of the viral genome [11]. The interaction between Tat and cyclin T1 requires zinc and a critical cysteine residue at position 261, and the lack of this residue (C261) greatly reduces the binding of Tat/p-TEFb complexes to TAR-RNA [5]. Acetylation of Tat at residues Lys28 and Lys50 is crucial for Tat function [12]. Mechanistically, acetylation at Lys28 by P/CAF enhanced Tat binding to the Tat-associated kinase, cdk9/p-TEFb, while acetylation by p300 at Lys50 of Tat promoted the dissociation of Tat from TAR RNA that occurs during early transcription elongation [13].

#### II- effect of HIV-1 proteins on the central nervous system

One of the most studied secondary manifestations of chronic HIV-1 infection is HAND (HIV-Associated Neurocognitive Disease). HAND encompasses a specific group of neuropathological conditions that emerge from the continued exposure of the CNS tissue to the HIV-1, HIV-1 viral proteins (Tat, Vpr, gp120, and Nef), immune inflammation and the combination of antiretroviral therapy (cART) [14]. HAND is generally divided into three main groups depending on the severity of the neurocognitive impairment as well as the impact on everyday lives of the infected individual: HIV-Associated Dementia (HAD) is the most severe form followed by Mild Neurocognitive disorder (MND) and the Asymptomatic Neurocognitive Impairment (ANI). Since the introduction of cART, HAD cases have decreased significantly, however more chronically infected individuals are diagnosed with the milder MND and ANI [15]. This can be attributed to the fact that in the cART era, while viral detection is at its minimum, in low penetration immune privilege system such as the nervous system, the transcription of viral proteins continues [16]. This leads to a constant cytotoxic stress, inflammatory response and tissue integrity damage, all of which are major contributors to HAND development and progression. Recently, it has been shown that viral replication might occur and could evade the innate immune recognition through the recruitment of (CPSF6) and cyclophilins (Nup358 and CypA) factors [17]. Due to its endless mutation, this new discovery might somehow explain the continuous shedding of viral proteins in the brain, even at minimum and undetectable level, leading to neuronal damage.

##### a- HIV-1 Tat and the BBB endothelial cells

**Figure 1:**
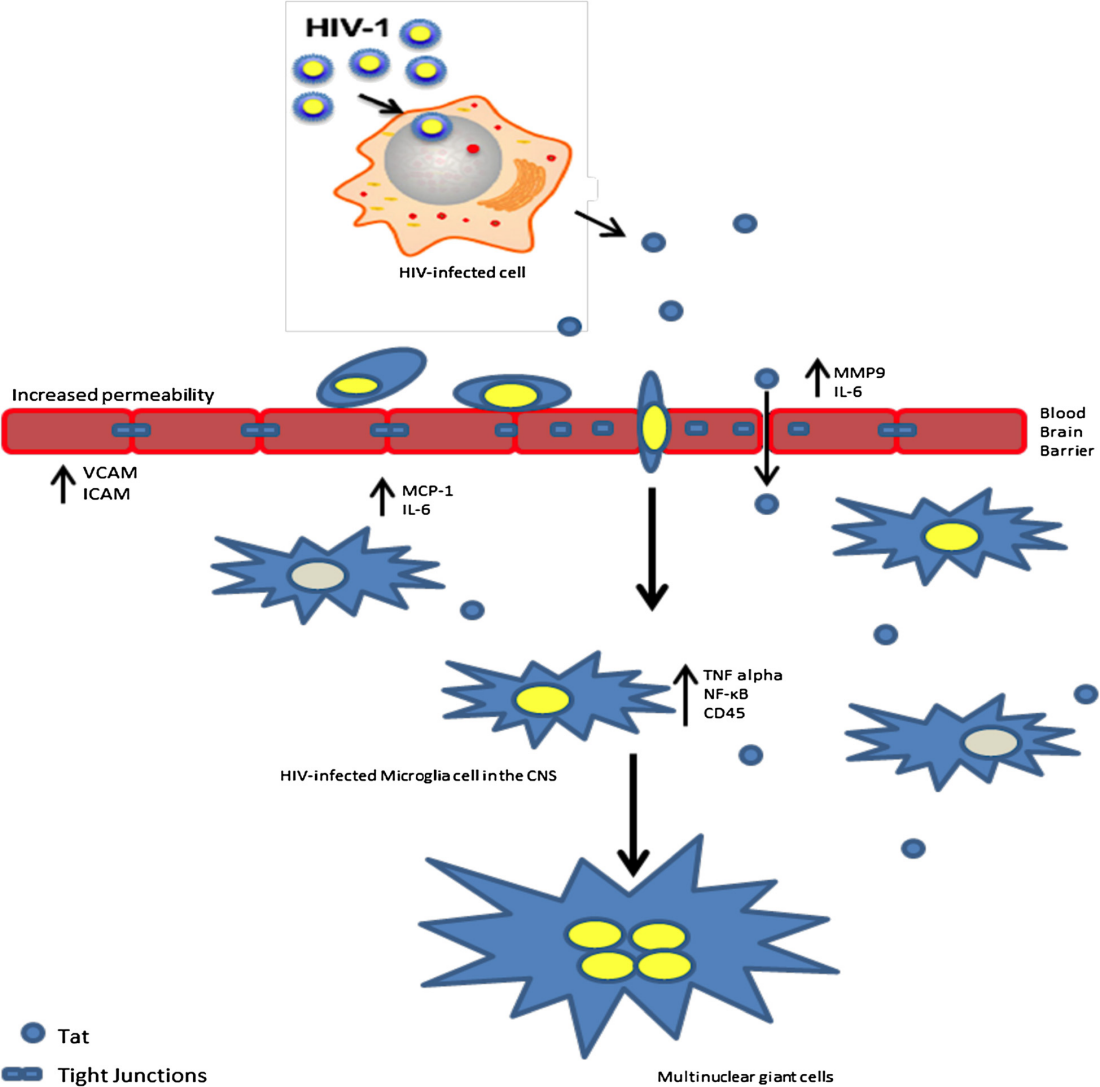
**Tat enters the brain through the BBB.** Schematic representation of Tat-modulation of the blood brain barrier. Some of the cellular factors involved are also shown.

HIV-1 infiltrates the brain soon after the initial infection (Figure 1). The initial “crossing” site of the virus is the Blood Brain Barrier (BBB). The BBB is composed of highly specialized monolayer of Brain Micro-Vascular Endothelial Cells (BMEC) lying on a relatively thick basal lamina. Astrocytes processes extend to the basal lamina and are in direct contact with it. They form a membrane structure that is supported by tight junctions between the cells. The integrity of the BBB is important for the support of brain homeostasis, since it has a selective permeability. It is a physical barrier to pathogenic agents such as bacteria and viruses and to large hydrophilic molecules, but is readily permeable to other small or hydrophobic molecules such as O_2_, hormones and CO_2_[18]. Several theories have been proposed that explain the mechanism of HIV-1 entry into the CNS. The “Trojan Horse” theory, a widely accepted, suggests that infected immune cells from the blood stream can accumulate and migrate through the BBB into the brain. Because of the inability of endothelial cells to be productively infected by HIV-1 [19], numerous deleterious vascular effects are thought to be mediated by secondary mediators, including the HIV-1-specific protein Tat. Moreover, cART treatment seems to have little or no effect on the secretion rate of Tat from infected cells in the CNS [20]. Since Tat can be released by infected monocytes and macrophages accumulated at the BBB [21], it can induce those changes either via receptor-mediated pathway or through a direct uptake of the protein in an active endocytosis manner [21,22]. Tat is responsible for changes in expression pattern of proteins important for the integrity of the endothelial tight junctions: claudins, occludins and junction adhesion molecules (JAMs). Those changes result in an increase permeability of the BBB [23-25]. Recent reports suggest that Tat protein is able to loosen up the tight junctions of the brain endothelial cells through occluding, by inhibiting it’s expression and cleaving it by matrix metalloproteinase 9 (MMP-9) [26]. In addition, Tat can trigger nuclear localization of ZO-1 via Rho signaling pathway which appears to be c-AMP response element-binding protein (CREB)-dependent response [20]. Interestingly, Tat is also able to penetrate a bi-lipid layer in a non-receptor transport mediated mechanism. This important characteristic of the protein is attributed to its transduction domain and it seems to be critical for the trans-endothelial transport of the protein [27-29]. More specifically, Cooper and his colleagues were able to show that CAYGRKKRRQRRR region of Tat is able to induce internalization of high molecular weights molecules [28]. All these studies have been predominantly performed *in vitro* using primary human brain microvascular endothelial cells (HBMECs) and astrocytes using Tat B peptide. It is known however that HIV-1C infected individuals are less likely to develop neurocognitive decline (see HIV-1 subtypes below). It seems that one reason for that is the reduced ability of Tat C to drive similar changes described above in the BBB as Tat B [24]. The ability of Tat to directly cross the BBB was also confirmed *in vivo* by Nath and colleagues [23]. They used radioactively labeled Tat (1–72) peptide injected intravenously. Interestingly, the areas of the mouse brain with highest permeably to Tat was the hippocampus, occipital cortex and hypothalamus, areas that are shown to be affected in Tat transgenic animals models as well in postmortem autopsies in HAND patients [30,31]. This ability of Tat to enter HIV-1 non-permissive cells could open new avenues for research not only in the context of HAND development but drug delivery as well. Tat protein is also considered to be an immune response activator. For example, in Tat treated endothelial cells, cAMP dependent protein kinase pathway is involved in protein kinase C dependent induction of IL-6 [32] which is associated with higher endothelial permeability.

##### b- HIV-1 Tat and Microglia

Once in the CNS, beyond the BBB, productive replication of HIV-1 can be supported by two cell types: microglia and the astrocytes. Microglia is a subtype of CNS immune cells that unlike the neuronal cells and astrocytes, which have neuro-ectoderm embryonic lineage, share the same origin as macrophages and other hematopoietic cells [33,34]. Previously, the involvement of this type of cells in brain diseases was largely seen as secondary to their progression. Currently, more evidence suggests the leading role that microglia cells play in brain pathologies including infections, facial nerve axotomy, Alzheimer's disease (AD), Parkinson's disease (PD), amyotrophic lateral sclerosis (ALS), HAND and stroke [35-37]. Microglia cells carry a specific role in the progression of HAND and Tat is shown to be cytotoxic and pro-inflammatory in the context of this pathological condition [38]. One of the physiological markers in advanced stages of HAND is microglial activation and multinuclear giant cells nodule formation. This can lead to changes in their immune effector functions, phagocytosis and pro-inflammatory signaling pathways such as TNF-alpha and beta-chemokine production [39,40]. Recently, novel leucine-rich repeat kinase 2 (LRRK2) was identified as a potential pharmaceutical target for microglia activation inhibitor [41]. Protein-tyrosine phosphatase (PTP), CD45 is another promising molecule, since it is an upstream target of the pro-inflammatory intracellular signaling mediators [42]. Additionally, IL-6 induction in microglia cells is NAPDH dependent and reversible by the use of specific inhibitors [43]. This correlates with recent data showing increase in the release of glutamate, a possible explanation of the neuronal hyper excitability mediated toxicity [44]. Cautious optimism in alleviating HAND symptoms brings the fact that Ibudilast, known non-selective cyclic AMP phosphodiesterase inhibitor, that has recently showed promise as a treatment for neuropathic pain via its ability to attenuate glial cell activation, also seems to attenuate Tat induction of the nuclear factor-kappa B (NF-κB) and TNF-alpha signaling activation [40,45]. Interestingly subtype C Tat protein was able to modulate the levels of tumor necrosis factor-receptor-associated factor 3 TRAF3 in a miR-32 dependent manner and can change the downstream expression of IRF3 and IRF7 [46]. The last finding might be an important insight, since both molecules are in the base of immune activation in response to various stimuli. Further, recently non-muscular myosin light chain kinase (nmMYLK) was described to be critical for microglial migration in Tat-treated cells and in Tat-transgenic mice, a phenomenon that is important during the innate immune response [47].

##### c- HIV-1 Tat and astrocytes

Unlike microglia cells, Astrocytes rise from the same neuro-ectoderm embryonic lineage as neurons [48]. They are in direct contact with neuronal cells and play critical supportive role in maintaining their homeostasis. Additionally, astrocytes have mechanical and signaling function in the formation of the Blood Brain Barrier (BBB) [49]. Although, astrocytes support productive HIV-1 infection in the CNS, they remain inaccessible to almost all known anti-retroviral treatments available [50]. Astrocytes are major contributor to the increased MCP-1 levels in the CNS in the context of HAND, Multiple Sclerosis (MS) and other neurodegenerative conditions as well [51-53]. Studies have demonstrated the ability of Tat protein to induce several genes in astrocystes such as MCP-1 through up-regulation of the (PDGF)-B [54,55]. Tat also activates the EGR-1 promoter via specific serum response sequences within the promoter. This could be interpreted as probably one of the upstream molecular events that initiate Tat-induced astrocyte dysfunction and subsequent Tat neurotoxicity [56,57]. As a target of MCP-1, Akt and Erk1/2 signaling proteins are indirectly affected by Tat. Additionally, reports suggest that Tat was able to induce COX-2 and PGE2 synthesis in astroglial cells through an NFAT/AP-1-dependent mechanism [58,59]. Another interesting finding demonstrates the importance of Tat cysteine-rich domain in regulating wnt/β-catenin signaling pathway. As a result wnt/β-catenin cascade is silenced [60,61]. This leads to abolishment of one of the natural HIV-1 transcriptional suppression mechanisms. Moreover, a clear difference was observed between subtypes B and C Tat proteins regarding their abilities to modify the wnt/β-catenin pathway.

Similar to microglia cells, HIV-1 Tat is associated with increased levels of nuclear factor-kappa B (NF-κB) in astrocytes as well, which in terms is linked to upregulation of adherence molecules such as vascular cell adhesion molecule-1 (VCAM-1) and intercellular adhesion molecule-1 (ICAM-1) [62,63]. Additionally, significant increases in TLR2 with reciprocal decreases in TLR9 expression in response to Tat are observed. This is usually associated with an increase in nitric oxide levels [64]. Interestingly, NADPH oxidase is responsible for HIV-1 Tat-induced generation of ROS and plays an important role in the up-regulation of adhesion molecules such as VCAM-1/ICAM-1 [63]. This is important since increased levels of those molecules correlates with increased adhesion of monocytes to astrocytes. These results suggest that Tat disrupts the innate immune response of the central nervous system (CNS) that may lead to increased pathogenicity.

Cell death cascade also appears to be activated as data suggest that the p53 family of proteins plays a suppressing role in HIV-1 Tat acetylation by P/CAF, a required interaction for activation of HIV-1 LTR promoter [65,66]. This appears to be mostly a defensive response to the HIV-1 infection, since it does not necessarily leads to astrocytes apoptosis.

Further, Tat has been shown to cause cell cycle disturbance through its interaction with p53 protein [67]. Extracellular Tat is rapidly internalized by neurons and astrocytes and this may inhibit p53 ubiquitination leading to p53 accumulation. Also, p53 accumulates in microglia and astrocyte nuclei in a subset of AIDS patients without dementia, while increased neuronal p53 was only observed in HAD cases ([65,66] and references within). Tat perform these functions through its physical interaction with p53 protein [68]. Finally, while Tat may physically associate with p53, it remains unable to activate p53 directly. Although, direct or indirect mechanisms, which lead to p53 activation in neurons in the context of HIV-1 remain unclear, it is highly likely that, like many other viruses, HIV-1 may stimulate p53 activation, which thereby alters the phenotype of uninfected microglia, and leads to neuronal loss. Note that p53 cannot induce apoptosis in response to DNA damage without p73 [66]. This illustrates that p73 is vital for p53-induced apoptosis and furthermore, that p73 is an important component of the tumor suppressor activity of p53. Finally, using astroglioma cell line, we recently demonstrated that HIV-Tat physically associates and induces the endogenous levels of p73, however, it inhibits its apoptotic activity and *vice versae*[65,66]. Tat induction leads to variety of different inclusions characteristic of lysosomes, autophagic vacuoles, and lamellar bodies, which were typically present within distal cytoplasmic processes that correlate with disrupted Long Term Potentiation (LTP) and memory formation in Tat-transgenic Mice [69].

##### d- HIV-1 Tat and oligodendrocytes

Oligodendrocytes are the myelin producing cells in the CNS. A single cell can maintain the myelin sheath of multiple neuronal cells, which is a key morphological characteristic for the proper function of the neuronal cell. Even though, this subtype of resident glial cells has not been shown to support any active HIV-1 infection, their crucial role suggests that any disruption in their function can potentially contribute to HAND progression. Although the biological significance *in vivo* is yet to be determined, it has been demonstrated *in vitro* that oligodendrocytes are susceptible to the HIV-1 Tat protein in a Caspase-3 dependent manner [70]. Further, progressive multifocal leukoencephalopathy (PML) is evident in HIV-1 infected individuals; however reports attributed that to be the result of a co-infection with a JC virus [71,72]. Recently, evidence emerges that the cART treatment might be further contributing to this condition as well [73,74]. Unfortunately, much more detailed studies are required to fully understand the effects of chronic HIV-1 infection and the various viral factors on oligodendrocytes in the CNS.

##### e- HIV-1 Tat and neuronal cells

Unlike microglial and astrocytes, neuronal cells does not support productive HIV-1 infection, however they experience a severe stress in the form of viral proteins, pro-inflammatory cytokines, disrupted BBB and cART [14,16,75,76]. Using Tat transgenic mice, studies have shown the deleterious effect that Tat has on the hippocampal, subcortical and cerebellum areas [4,30]. Those pathophysiological changes are not only the results of astrocytes and microglia deregulation, but Tat protein can directly affect the function and viability of neurons as well. Two main theories emerge from the literature about the mechanism of direct Tat neurotoxicity: 1) Tat is able to induce changes in the neuronal cell homeostasis via extracellular signaling mechanism including receptors, changes in membrane permeability and composition; 2) Internalization of Tat protein leads to direct interaction with cellular factors involved with Ca^2+^ regulation, transcription and translation.

Tat has been extensively studied in *in vitro* systems and has been shown to increase Ca^++^ flux and to impair synaptic plasticity leading to neuronal deregulation. NMDA receptor for example catches often the researcher’s attention, because of its key importance in Ca^++^ regulation and membrane polarization [77]. Recent studies suggest that neuronal cell susceptibility relies on the expression levels of NMDAR. Not only that, but different subunits appear also to exacerbate the effect of Tat. For example rat hippocampal neurons appear less susceptible to Tat even though they highly express NMDA receptor. This might be related to low levels of NR2A subunit [78]. Moreover, in differentiated human primary neurons, Tat is able to promote the phosphorylation of Tyr1184, 1325, and Tyr1425 within the NR2A subunit [79].

Another example supporting the extracellular receptor mediated signaling theory is the growing evidence that indicates that HIV-1 Tat protein may affect the function of the dopamine transmission system. In turn, molecular components of dopamine neurotransmission may participate in a complex network of Tat-induced cell responses that result in neurodegenerative conditions. It appears that Tat induced-neurotoxicity has a reverse correlation with the D1 dopamine receptor expression levels and function. Higher levels of D1 will be related to lower apoptosis and blocked function of D1 to increased apoptosis rate in Tat-treated cells [80,81]. Interestingly, Tat inhibits dopamine (DA) transporter (DAT) function through a PKC and trafficking-dependent mechanism and that Tat impacts the dopaminergic tone by regulating both DAT and vesicular monoamine transporter (VMAT2) proteins [82]. Those changes, at least in DAT function are related to activation of ryanodine receptor (RyRs) via a calcium- and calpain-mediated mechanism, and is independent of DAT protein synthesis, reinforcing the feasibility of RyR and GSK-3β inhibition as clinical therapeutic approaches for HAND [83]. However, the question to which specific RyRs are responsible for this signaling cascade is open for discussion. These findings further provide insight into understanding the mechanisms of HIV-1 viral protein-induced dysfunction of DA neurotransmission in HIV-1 infected patients.

In addition to cell surface receptor mediated signaling, HIV-1 Tat has the unique property of entering the cell in a calveolar and lipid rafts dependent manner. This property of the Tat protein is widely used for mediating the delivery of large protein cargos into cells when present in the extracellular milieu [84]. However, it seems like that this is an understudied area in the subfield of Tat induced-neurotoxicity. In BBB endothelial cells for example, Tat treatment leads to elevated GTP-RhoA levels and its downstream effectors, such as myosin phosphatase target subunit 1 and myosin light chain. In addition, Tat upregulates expression and promoter activity of *P*-*gp* gene as well as its efflux function. Inhibition of the Rho signaling cascade effectively blocked P-gp overexpression at the level of promoter activity. Disruption of lipid rafts by depletion of membrane cholesterol by methyl-beta-cyclodextrin, but not caveolin-1 silencing, also abolished Tat-mediated RhoA activation and P-gp upregulation [85]. This shows the critical function of intact lipid rafts and the Rho signaling in HIV-1 Tat-mediated upregulation of P-gp at least in endothelial cells even though it plausible that similar model might be applied to neuronal cells as well. Additional reports suggest that Tat uses numerous receptor- mediated pathways including CD26, CXC chemokine receptor type 4(CXCR4), heparin sulphate proteoglycans and LDL (low-density lipoprotein) receptor-related proteins [86-89]. This also suggests an active endocytosis mediated entry of HIV-1 Tat in neurons. Endocytosis is a fundamental function that plays critical role for the maintenance of neuronal function [90].

Recently, endolysosome pathway has been implicated in a variety of neurological disorders including AD (Alzheimer's disease), Parkinson's disease and HAND [91,92]. HIV-1 Tat can accumulate in endolysosomes, which leads to endolysosomes size increased, membrane integrity disruption, pH elevation, and autophagy inhibition. Once inside the cells, the protein can be released in the cytoplasm and latter translocated in the nucleus [93]. Moreover, Tat peptide contains binding motifs for two specific protease enzymes: Furin and Calpain (Table 1). Interestingly, both enzymes are Ca^++^ dependent and while Furin cleavage leads to inactivation of Tat function as LTR activator, Clapain cleavage leads to increased neurotoxic activity. It is an open question whether truncation of Tat leads to increased neurotoxic activity and if so, which parts of the peptide are responsible.

**Table 1:** **Cellular factors cleaving Tat (**http://www.ncbi.nlm.nih.gov/projects/RefSeq/HIVInteractions/**)**

**Protein name**	**Protein Acc**	**Ref PMID**	**Interaction description**
Calpain-1 catalytic subunit	NP_005177.2	19022302	Calpain-mediated cleavage of HIV-1 Tat occurs in the C-terminus of this viral protein, between amino acids 68 and 69. The cleavage of Tat by calpain 1 increases neurotoxic effect of this viral protein
Furin preproprotein	NP_002560.1	15135058	Furin cleaves HIV-1 Tat at amino acid residue 56, resulting in greatly reduced Tat transactivation activity

Tat is associated with higher levels of nuclear and mitochondrial genomic DNA damage in the brain. High level of nuclear and mtDNA 8-oxoG damages were identified in the cortex autopsy tissue of HAND patients. Increased accumulation of mtDNA mutations and/or depletion was also detected to occur in brain tissue in a subset of HAND individuals [94]. However, these results do not discriminate between different cell types in the brain and further validation is required. Additionally, HIV-1 Tat can cause a rapid dissipation of the mitochondrial transmembrane potential, as well as cytochrome *c* release in isolated mitochondria. Pharmacological studies reveal that Tat induces mitochondrial membrane permeabilization (MMP), which is Bax/Bak, Bcl-2 and Bcl-x_L_ independent and can be rescued by the anion-channel inhibitor 4,4’-diisothiocyanostilbene-2,2’-disulfonic acid (DIDS), but not by the ruthenium red, or ryanodine receptor blocker. Moreover, Tat is able to inhibit the cytochrome c oxidase (COX) activity in disrupted mitochondria making it the first viral protein to be a plausible COX inhibitor [95]. All this is an indicator that Tat can induce mitochondrial dysfunction in neurons independently from the Ca^++^ flux and receptor mediated pathways previously described. Taking into account the central role that mitochondria have in neuronal function, it is easy to see why neuronal cell exhibit high susceptibility to HIV-1 Tat protein. Another important aspect is the synaptic and axonal injury observed in HAND individuals and in Tat transgenic mice as well. In the context of HAND, synaptic loss can be a defense mechanism that allows the neuronal cells to deal with the over excitatory conditions in the CNS [96]. On the other hand it can be a consequence of the physical infiltration of phagocytic leucocytes, which directly target for destruction synapse endings [97]. Although some studies suggest that Tat induced synaptic loss is Ca^++^ dependent via NMDAR and low-density lipoprotein receptor-related protein (LRP), the molecular mechanism involved is not quite clear [98]. Release of dopamine, glutamate, GABA and acetylcholine neurotransmitters are all shown to be affected by Tat protein and it can lead to diminished LTP, induction of LTD or even an impaired signal transduction between neuronal cells [99-101].

Recent studies reveal that some of the pathways already described to be involved in neuronal deregulation are affected by expression levels of micro RNA molecules (miRNAs). miRNAs introduce a novel concept of regulatory control over gene expression, and there is increasing evidence that they may play a profound role in neuronal cell identity as well as multiple aspects of disease pathogenesis [102]. In support of this observation, a link between miRNAs and neurodegenerative diseases (*e.g*. Alzheimer, Huntington, and Parkinson) is becoming increasingly evident [103,104]. Interestingly, Tat treatment of primary human neurons leads to alteration of the expression profile of miRNAs which in term affects critical pathways required for the neuronal cell function [4]. For example, miRs-1, -7, and -34a were examined and validated to deregulate the levels of SERP1 (stress-associated endoplasmic reticulum protein 1 involved in Endoplasmic Reticulum stability), Drp-1 (Dynamin-related protein nvolved in proper mitochondria distribution among the neuronal cell), as well as CREB (a key Ca^++^ dependent transcription factor in LTP/LTD). The importance of CREB role was also described to be a part of the Pyk2/Erk/CREB pathway, where TRPC channels have been shown to prevent Tat toxicity by inducing the Platelet-derived growth factor-BB (PDGF) [105].

##### f- HIV-1 Tat outside the CNS additional contributing factors, concerns and alternatives

The deleterious effect of Tat in CNS is not without a precedent and has been shown in different organs. In this regard, it has been shown, that the effect of Tat on osteoclast/ osteoblast crosstalk and homeostasis. HIV-1 infected individuals often suffer from secondary bone remodeling conditions osteopenia/osteoporosis. Key estrogenic factors such as RANKL and M-CSF are deregulated in Tat dependent manner, which leads to hyperactive osteoclast [106].

Another organ system affected is the Urinary system. In the context of HIV-Associated Nephropathy, Tat protein increased albumin permeability and rapidly induced the redistribution and loss of nephrin in isolated glomeruli [107]. Furthermore, studies investigating other HIV-Associated conditions such as HIV-Associated Thrombocytopenia, Pulmonary vesicular remodeling as well as Cancer development suggest that HIV-1Tat protein plays significant role in the pathological progression of those diseases [108-111].

HIV-1 Tat is only one of the many contributing factors that lead to HIV neurocognitive impairment progression. Gp120, Vpr and Nef proteins are all shown to affect normal neuronal function, neurotropic molecules release and immune activation. Gp120 for example is shown to up-regulate Matrix metalloproteinases (MMPs) 2 and 9 which highly correlates with Blood Brain Barrier disruption [112]. VPR is able to induce neuronal cells death and Nef is linked to spatial and recognition memory lost by targeting specifically CA3 Hippocampal neurons in *in vivo* mouse models [113,114]. One of the pushbacks whenever investigating the effect of viral proteins in vitro is the use of viral protein concentrations that does not necessarily correspond to physiological conditions in the CNS. This is very critical and one should be extremely careful how to interpret results from in vitro neuronal cells experiments, since there is no active production of viral proteins in this cell type. Studying abnormal function in Astrocytes and Microglia cells, which in most cases include overexpression approach, is much more physiologically relevant since these cell types have been shown to support active HIV-1 infection and viral proteins release. This is why in vitro studies involving viral proteins and neuronal cells should be always validated using appropriate transgenic mice and co-culture assays, which represent more physiologically similar conditions.

Another aspect in studying factors contributing to neurocognitive impairment in HIV-1 positive individuals is the link between the evident immune activation and co-existing predisposing conditions such as alcohol and drugs of abuse.

##### g- impact of Tat on different subtypes

Analyses of different strains of HIV-1 show that isolates can be subdivided into groups, subtypes, and circulating recombinant forms (CRFs), based on phylogenetic sequence differences. So far, HIV-1 can be divided into three distinct and highly divergent groups: M (major), O (outlier), and N (non-M/O). Several genetic variants can be recognized within group M, including 9 subtypes/clades and 15 major CRFs [115,116]. Subtype C predominates globally and, in the year 2000, caused 47.2% of all new HIV-1 infections. The second most common clade was A, which caused 30% of all new infections, (including CRF01-AE and CRF02-AG) [115]. Clade D is generally limited to Eastern and Central Africa, E appears as an A/E mosaic detected in South and East Asia [117-120]. F has been reported in Central Africa, South America and Eastern Europe. G and A/G recombinant viruses have been observed in Western and Eastern Africa as well as in Central Europe [120]. H and K have only been detected in central Africa [121-123]. J has been reported in Central America and Central Africa [124].

Since B is the predominant subtype in the western world, antiretroviral drugs used to treat HIV were developed using *in vitro* studies of subtype-B isolates, and most data on HIV-1 drug resistance mechanisms are from subtype-B viruses. Nevertheless, subtype B is responsible for only 12% of global infections [116]. This leads to the question whether or not HIV-1 subtypes do have any consequences on therapy outcome and the development of drug resistance, especially in regards to the implementation of antiretroviral drugs in areas with high non-B subtypes. Further, one may wonder whether subtype variations affect disease progression. For example, several groups demonstrated that, due to some unknown reasons, patients infected with HIV-1 clade C manifested less neurocognitive disorders than those infected with clade B.

The problem of subtype diversities is becoming more significant in the western world [125], especially with increasing migration and globalization where at least 25% of new infections in Europe are presently non-B African and Asian variants. The relationship between HIV-1 subtype diversities, disease transmissibility and progression is poorly understood especially regarding development of neurocognitive disorders (HAND) that display in the brain as the “white matter disease” due to neuronal degeneration and loss. Even though introduction of HAART diminishes the incidence rates for HIV dementia in the USA where HIV-1 clade B is the predominant genotype however, with continued survival, the prevalence of this disorder has actually increased. All these signify the importance for the development of new therapeutic measures, which will also address the HIV-1 subtype diversities.

Since inter-subtype studies may be complicated by host, sequence variations, societal and virological factors that are difficult to control, several groups suggest that AIDS progression differs as a function of infecting subtype [126,127]. Their results focused mainly on polymerase (Pol), reverse transcriptase (RT), envelope (gp120, gp41) and the promoter (LTR) sequences variations. Variations in these regions may therefore affect drug susceptibility and development of drug resistance [128,129]. For example Ethiopian clade C isolates differ (with respect to RT) from clade B by 6.8-10%. Also intra-clade differences of 3.5–5.8% have been reported for strains from Africa, India and South America [127]. It should be stressed that any given percentage variation in nucleotide sequence translates into lower amino acid sequence variation is notable because many genetic mutations are silent. Additionally, it has been shown that the LTR sequences, which contain transcriptional promoters of HIV-1 vary substantially from clade to clade [130]. Each clade has its own LTR copy number as well as an exact nucleotide sequence of enhancer and promoter structures, despite the uniformity in other LTR features, i.e. Sp1 sites, TATA box and TAT-responsive element [131,132]. However, diversity was observed in numbers of transcriptional promoters. These include the NF-κB binding sites (3 to 4 in C, 2 in B and just 1 in E), the AP-1 transcriptional factor-binding site (1 site in subtypes C, E and G, 2 in A and F, and none in B or D) and the C/EBP-β binding-site (exists only in clade B but not in A or C). Further, subtype discrepancies arise also between the negative regulatory element seen in clades C and E versus that detected in clade B [133]. Given these genetic distinctions between HIV-1 promoters, it is not surprising to find that clades respond differentially to various transcriptional factors. For example, the NF-κB transcription factor stimulates HIV-1 clade C to a far greater extent than clade B or E [134,135]. Likewise, tumor necrosis factor (TNF-α) activates the LTRs of clade C more impressively than those of clades A, B, D, F and G, with the lowest stimulation seen in clade E [136]. Thus, one might suggest that these intra-sequence variations might implicate the recruitment of different transcription factors through the recruitment of diverse clusters of small RNAs that regulate expression of these factors. If so, these RNAs also play a role in HIV-1 latency and disease progression leading to HAND.

Sequence variations were also observed within the viral genes (e.g. Tat). The transactivator regulatory protein (Tat) plays a major role in viral gene expression and replication. In addition, it has been described to be involved in the process of disruption of neuronal function [137]. Several studies examined the functions of Tat prepared from different clades especially since significant amino acid variations have been observed among the clade-specific Tat proteins. It has been shown that acetylation of subtype-specific Tat proteins may correlate with their transactivation efficiency [136]. Further, Tat proteins derived from HIV-1 clades C and E were strong transactivators of the HIV- promoter compared to other Tat proteins from clades A and B [138]. Tat was also used as a candidate for vaccine. Studies showed that macaques immunized with clade B Tat developed strong antibody responses when compared to Tat prepared from clade C [139]. Moreover, in mice, cross-clade immune responses between HIV-1 subtype B and C Tat proteins mapped to this T-helper epitope was identified [136]. These results led to the conclusion that a cross-clade immune response between subtypes B and C is important for a more rational design of an HIV vaccine. This conclusion was later confirmed when Tat B-clade was similarly recognized by sera from individuals infected by different virus clades A, B, C and D supporting the concept of a cross-clade vaccine [140]. Finally, using primary human neurons, subtype C Tat was shown to be less toxic than subtype B Tat [141]. In addition, clade B Tat protein was shown to increase the level of neuropathogenic agents, such as IDO and KYN in human primary astrocytes when compared to clade C Tat [142]. Taken together, these studies provide further evidence that the prevalence of HAND may be correlated with the difference in clades of HIV-1 especially since Tat has been shown to be involved in AIDS neuropathogenesis.

### Concluding remarks

Thirty years of HIV-1 research have led to great advances in the control and treatment of the infection, but there still is a long way ahead in the quest of controlling the virus. The new generation of cART allows HIV-1 infected patients to live longer, almost normal life expectancy lives. Unfortunately secondary complications from the chronic infections and even the drugs themselves are degrading the quality of live for these individuals. Understanding in greater details how the virus and its proteins affect the cells on a basic molecular level will greatly increase the opportunity to design proper defensive strategies that will allow us to alleviate those pathologies. This review summarizes the advancements in only one aspect of the problem: HIV-1 Tat protein. It is surely one of the many factors in the big picture of HIV-1 infection, but as research suggests it is a very important one. With its unique ability to travel between cells and affect fundamental pathways that are important for the proper function of the cell, Tat reveals the very complex and unconventional network of tasks that this protein caries and the potential for future research. The findings from such a research can be applied not only in the field of HIV-1, but in other biological areas as well. One example today is the use of Tat peptides to drive different size molecules internalization. This property can be used in designing new strategies for drug deliveries even beyond the blood brain barrier. The role of small noncoding RNA species in the progression of the disease is another promising topic since there is an increasing body of research describing their fundamental importance in the neurodevelopment. In conclusion we have listed several tables with cellular factors important for Tat functions both canonical and secondary containing endogenous proteins that interact synergistically (Table 2) and cellular factors described to be functionally involved (Table 3) with Tat in general.

**Table 2:** **Cellular factors interacting synergistically with Tat (**http://www.ncbi.nlm.nih.gov/projects/RefSeq/HIVInteractions/**)**

**Protein name**	**Protein Acc**	**Ref PMID(s)**	**Interaction description**
Beta-nerve growth factor precursor	NP_002497.2	8178451	Nerve growth factor synergizes with HIV-1 Tat to induce HIV-1 gene expression in neuronal and glial cell lines
CD40 ligand	NP_000065.1		Recombinant CD40L synergizes with HIV-1 Tat to increase TNF-alpha release from primary human monocytes and microglia in an NF-kappaB-dependent manner. This synergism is attributed to a Tat-mediated up-regulation of CD40
CDK-activating kinase assembly factor MAT1 isoform 1	NP_002422.1	8934526	TFIIH synergizes with HIV-1 Tat to induce transcription elongation from the HIV-1 LTR promoter
Cyclin-dependent kinase 7	NP_001790.1		
Cyclin-H isoform 1	NP_001230.1	8934526	
Cytochrome B-245 heavy chain	NP_000388.2	21029719	Nox2 is involved in HIV-1 Tat-induced NADPH oxidase p65 and IKK phosphorylation
E3 SUMO-protein ligase EGR2 isoform a	NP_000390.2	11909874	HIV-1 Tat (through amino acids 30–40) binds to Egr-2 and synergizes with this protein to super induce the FasL promoter
Early growth response protein 3 isoform 1	NP_004421.2		
Endothelial transcription factor GATA-2 isof. 1	NP_116027.2	9517987	GATA-2 synergizes with HIV-1 Tat to enhance transcriptional activity from the HIV-1 LTR promoter
Fibroblast growth factor 2	NP_001997.5		HIV-1 Tat synergizes with bFGF to promote Kaposi's sarcoma, endothelial cell growth and locomotion, and secretion of matrix-metalloproteinase-2
General transcription factor IIF subunit 1 & 2	NP_002087.2		TFIIF synergizes with HIV-1 Tat and the cellular co-activator Tat-SF1 during Tat-mediated transactivation of the HIV-1 LTR promoter
	NP_004119.1		
General transcription factor IIH subunit 1, 2, 3 and 4	NP_005307.1	8934526	TFIIH synergizes with HIV-1 Tat to induce transcription elongation from the HIV-1 LTR promoter
	NP_001506.1		
	NP_001507.2		
	NP_001508.1		
Histone acetyltransferase KAT2B	NP_003875.3		HIV-1 Tat synergizes with P/CAF to activate the HIV-1 LTR promoter
Histone acetyltransferase p300	NP_001420.2	18226242	HIV-1 Tat, NAP-1, and p300 synergistically activate HIV-1 transcription
Interferon gamma precursor	NP_000610.2		IL-1beta, TNF-alpha, and IFN-gamma synergize with HIV-1 Tat to promote in nude mice the development of angioproliferative Kaposi's sarcoma-like lesions
		10446807	HIV-1 Tat synergizes with IFN-gamma to induce iNOS activity in purified rat microglial cultures
		18569454	Tat and IFN-gamma synergistically induce the expression of CXCL10, which is inhibited by MEK1/2 inhibitor and the p38 mitogen-activated protein kinase (MAPK) inhibitor
			HIV-1 Tat in combination with IFN-gamma and TNF-alpha increases CXCL10 mRNA and protein in human astrocytes through the activation of the p38, Jnk, and Akt signaling pathways and their downstream transcription factors, NF-kappaB and STAT-1alpha
		19941336	HIV-1 Tat increases CXCL10 expression in IFN-gamma and TNF-alpha stimulated human astrocytes via NADPH oxidase
Interleukin-1 beta protein	NP_000567.1	10438928	IL-1beta, TNF-alpha, and IFN-gamma synergize with HIV-1 Tat to promote in nude mice the development of angioproliferative Kaposi's sarcoma-like lesions
Nuclear factor 1C-type isoform 5	NP_005588.2		HIV-1 Tat synergizes with CTF to activate transcription and enhance transcript elongation and exon skipping
Nuclear factor of activated T-cells, cytoplasmic 1 isoform A	NP_765978.1		NFATc synergizes with NF-kappa B and HIV-1 Tat in transcriptional activation of the HIV-1 LTR promoter and enhances HIV-1 replication in T cells
Nucleosome assembly protein 1-like 1	NP_004528.1	18226242	HIV-1 Tat, NAP-1, and p300 synergistically activate HIV-1 transcription
RISC-loading complex subunit TARBP2 isoform a	NP_599150.1		TRBP2 binds to HIV-1 TAR RNA and synergizes with HIV-1 Tat to activate the HIV-1 LTR promoter
TATA-box-binding protein isoform 1	NP_003185.1		TBP synergizes with HIV-1 Tat during Tat-mediated transactivation of the HIV-1 LTR promoter
T-box transcription factor TBX21	NP_037483.1	18300036	Pretreatment of THP-1 cells with HIV-1 Tat/T-bet co-cultures with CD4 + T cells, resulting in increased levels of IFN-gamma
TFIIH basal transcription factor complex helicase XPB subunit	NP_000113.1	8934526	TFIIH synergizes with HIV-1 Tat to induce transcription elongation from the HIV-1 LTR promoter
TFIIH basal transcription factor complex helicase XPD subunit isoform 1	NP_000391.1		
Thyroid hormone receptor alpha isoform 2	NP_003241.2		HIV-1 Tat synergizes with thyroid hormone (T3) receptor alpha to activate the HIV-1 LTR promoter in the absence of T3, which is relieved in its presence, suggesting a possible role for T3 in the control of HIV-1 gene expression
Transcription elongation factor A protein 1 isoform 1/ protein 2 isoform α/ protein 3	NP_006747.1	1559613	TFIIS synergizes with HIV-1 Tat during transactivation of the HIV-1 LTR promoter
	NP_003186.1		
	NP_003187.1		
Transcription factor Sp1 isoform a			Sp1 synergizes with HIV-1 Tat to activate HIV-1 transcription
Tumor necrosis factor		15246652	HIV-1 Tat synergizes with TNF-alpha to enhance IL-6 secretion and activate human central nervous system-derived endothelial cells
		19479051	HIV-1 Tat synergizes with TNF-alpha to induce the expression of adhesion molecules ICAM-1, VCAM-1 and ELAM-1
		19941336	IL-1beta, TNF-alpha, and IFN-gamma synergize with HIV-1 Tat to promote in nude mice the development of angioproliferative Kaposi's sarcoma-like lesions
			HIV-1 Tat and TNF-alpha synergistically activate the adhesion of leukocytes to endothelial cells
			HIV-1 Tat in combination with IFN-gamma and TNF-alpha increases CXCL10 mRNA and protein in human astrocytes through the activation of the p38, Jnk, and Akt signaling pathways and their downstream transcription factors, NF-kappaB and STAT-1alpha
			HIV-1 Tat increases CXCL10 expression in IFN-gamma and TNF-alpha stimulated human astrocytes via NADPH oxidase
Zinc finger protein GLI2	NP_005261.2	11160734	GLI-2 physically interacts with HIV-1 Tat (demonstrated in GST pull-down experiments) and strongly synergizes with Tat during transactivation of the HIV-1 LTR promoter

**Table 3:** **Cellular factors binding to Tat (**http://www.ncbi.nlm.nih.gov/projects/RefSeq/HIVInteractions/**)**

**Protein name**	**Protein Acc**	**Ref PMID**	**Interaction description**
Activated RNA polymerase II transcriptional coactivator p15	NP_006704.3		Through amino acids 22–91, PC4 binds to the basic TAR binding domain of HIV-1 Tat (amino acids 49–57) and enhances activation of the HIV-1 LTR promoter in a Tat dependent manner
Adenylate kinase isoenzyme 6	NP_003178.1		HIV-1 Tat binds, through amino acids 36–50, directly to the TBP subunit of the TFIID holoenzyme complex (which includes at least TFIID subunits p250, p125, p70, TBP, and p30), and increases the interaction of TFIID with the HIV-1 LTR promoter
Aggrecan core protein isoform 1 precursor	NP_001126.3		HIV-Tat peptide interferes with polyamine uptake via competition for proteoglycan binding sites rather than a putative downstream transporter in human carcinoma cells
AT-rich interactive domain-containing protein 1A isoform a	NP_006006.3		Acetylated HIV-1 Tat binds efficiently to BRG1 and BAF200 (component of PBAF complex) and weakly to BAF250 (component of BAF complex). BAF250 has a preference to bind to unmodified Tat
AT-rich interactive domain-containing protein 2	NP_689854.2		Acetylated HIV-1 Tat binds efficiently to BRG1 and BAF200 (component of PBAF complex) and weakly to BAF250 (component of BAF complex)
B-cell lymphoma/leukemia 11B isoform 1	NP_612808.1		CTIP2 harbors two HIV-1 Tat interaction interfaces (amino acids 145–434 and 717–813) and binds to the N-terminus (amino acids 1–48) of Tat
Bone marrow proteoglycan isoform 1 preproprotein	NP_002719.3		HIV-Tat peptide interferes with polyamine uptake via competition for proteoglycan binding sites rather than a putative downstream transporter in human carcinoma cells
C-C chemokine receptor type 2 isoform A	NP_001116513.2		HIV-1 Tat binds to CCR2 and displaces MCP-1 from this beta-chemokine receptor, an effect mediated by Tat amino acids 24-51
C-C chemokine receptor type 3 isoform 1	NP_001828.1		
CCAAT/enhancer-binding protein beta	NP_005185.2	9169458	HIV-1 Tat induces an increase in C/EBPbeta binding activity through a direct binding interaction between Tat and C/EBPbeta that is mediated through the N-terminal, cysteine rich, and core regions of Tat (amino acids 1–47)
CDK-activating kinase assembly factor MAT1 isoform 1	NP_002422.1		Amino acids 1–48 of HIV-1 Tat, which includes the Tat activation domain, mediate the binding of Tat to CAK and the TFIIH complex through a direct interaction with CDK7 and possibly other TFIIH subunits, including p62 and ERCC3
Cellular tumor antigen p53 isoform a	NP_000537.3		HIV-1 Tat binds to p53, an interaction mediated by the basic region of Tat (amino acids 49–57) and the acidic O2 domain of p53 (amino acids 341-354
	NP_000537.3		The p53 tetramerization domain (residues 326–355) binds directly to residues 1–35 and 47–57 in HIV-1 Tat as evidenced by using peptide mapping, fluorescence anisotropy, and NMR spectroscopy
Complement component 1 Q subcomponent-binding protein, mitochondrial precursor	NP_001203.1	7778269	Using a yeast two-hybrid system, the splicing factor SF2-associated protein p32 has been shown to bind to the basic domain of HIV-1 Tat (amino acids 47–59), suggesting a role for p32 in mediating the biological activity of Tat during HIV-1 replication
	NP_001203.1		Splicing factor SF2-associated protein p32 preferentially binds acetylated HIV-1 Tat and co-localizes with Tat in HIV-1 infected cells
Core histone macro-H2A.1 isoform 1	NP_613075.1		HIV-1 Tat peptides bind core histones H2A, H2B, H3 and H4, and Tat protein recruits histone acetyltransferases to the HIV-1 LTR promoter leading to acetylation of histones H3 and H4, de-repressing chromatin structure and increasing NF-κB responsiveness
Core histone macro-H2A.2	NP_061119.1		
CREB-binding protein isoform a	NP_004371.2		HIV-1 Tat binds to the minimal histone acetyltransferase domain of the CBP/p300 complex (amino acids 1253–1710 of p300) and E1a binding domain (amino acids 1542–1710) of p300, an effect mediated by the basic domain (amino acids 48–57) of Tat
			The N-terminal 24 amino acids of HIV-1 Tat mediate its binding to the KIX domain (amino acids 589–679) of CBP
C-X-C chemokine receptor type 4 isoform b	NP_003458.1		HIV-1 Tat binds to CXCR4, competes with the natural ligand of CXCR4, SDF-1alpha, and selectively inhibits the entry and replication of ×4-tropic HIV-1 in peripheral blood mononuclear cells (PBMCs), indicating a role for Tat in selecting against ×4 virus
Cyclin-dependent kinase 2 isoform 1	NP_001789.2		HIV-1 Tat 41/44 peptide TAALS from the core domain of Tat inhibits Tat-mediated HIV-1 gene expression and replication by binding the Cdk2/Cyclin E complex and inhibiting the phosphorylation of serine 5 of RNAPII
Cyclin-dependent kinase 7	NP_001790.1		Amino acids 1–48 of HIV-1 Tat, which includes the Tat activation domain, mediate the binding of Tat to CAK and the TFIIH complex through a direct interaction with CDK7 and possibly other TFIIH subunits, including p62 and ERCC3
			TFIIH subunits CDK7 and cyclin H have been identified as two components associated with the Tat-associated CTD kinase (TTK) that binds to HIV-1 Tat
Cyclin-dependent kinase 9	NP_001252.1	7853496	The N-terminus (amino acids 1–48, including activation domain) of HIV-1 Tat binds to P-TEFb through a direct interaction with the N-terminus (amino acids 1–290) of cyclin T1 during Tat-mediated transactivation of the HIV-1 LTR promoter
		8676484	
		9356449	
Cyclin-H isoform 1	NP_001230.1		Amino acids 1–48 of HIV-1 Tat, which includes the Tat activation domain, mediate the binding of Tat to CAK and the TFIIH complex through a direct interaction with CDK7 and possibly other TFIIH subunits, including p62 and ERCC3
	NP_001230.1		TFIIH subunits CDK7 and cyclin H have been identified as two components associated with the Tat-associated CTD kinase (TTK) that binds to HIV-1 Tat
Cyclin-T1	NP_001231.2	7853496	The N-terminus (amino acids 1–48, including activation domain) of HIV-1 Tat binds to P-TEFb through a direct interaction with the N-terminus (amino acids 1–290) of cyclin T1 during Tat-mediated transactivation of the HIV-1 LTR promoter
		8676484	
		9356449	
Cyclin-T2 isoform a	NP_001232.1		Amino acids 260–263 of cyclin T1 are critical for HIV-1 Tat-mediated transcriptional activation, and site-directed mutations in this region of cyclin T2 (asparagine to cysteine at residue 260) allow it to bind Tat and stimulate transcription
Dipeptidyl peptidase 4	NP_001926.2		The N-terminal nine amino acids of HIV-1 Tat mediate the binding of Tat to CD26
DNA-dependent protein kinase catalytic subunit isoform 1	NP_008835.5	9525578	Amino acids 56–101 of HIV-1 Tat mediate Tat binding to DNA-PK, an effect that augments DNA-PK-mediated phosphorylation of Sp1 during Tat transactivation of the HIV-1 LTR promoter
E3 ubiquitin-protein ligase TRIM32	NP_036342.2	7778269	HT2A specifically and precisely binds to the activation domain of HIV-1 Tat (amino acids 1–48), suggesting a role for HT2A in mediating the biological activity of Tat during HIV-1 replication
Early growth response protein 1	NP_001955.1		HIV-1 Tat binds to Egr-1, an interaction that is mediated through Tat amino acids 30-40
G1/S-specific cyclin-E1	NP_001229.1		HIV-1 Tat 41/44 peptide TAALS from the core domain of Tat inhibits Tat-mediated HIV-1 gene expression and replication by binding the Cdk2/Cyclin E complex and inhibiting the phosphorylation of serine 5 of RNAPII
G2/mitotic-specific cyclin-B1	NP_114172.1		HIV-1 Tat stimulates polyubiquitination-mediated degradation of cyclin B1 through binding to the N-terminal of cyclin B1 (amino acids 61–129) that is just downstream of the D box
General transcription factor IIH subunit	NP_005307.1		Amino acids 1–48 of HIV-1 Tat, which includes the Tat activation domain, mediate the binding of Tat to CAK and the TFIIH complex through a direct interaction with CDK7 and possibly other TFIIH subunits, including p62 and ERCC3
Granulins precursor	NP_002078.1		The cysteine rich region of HIV-1 Tat (amino acids 21–37) mediates the binding of Tat to granulin amino acids 206–337 (granulin regions B + A) suggesting a role for granulin growth factors as biologically important extracellular Tat co-factors
Growth factor receptor-bound protein 2 isoform 1	NP_002077.1		The binding between HIV-1 Tat and Grb2 is mediated by the proline-rich sequence (residues 1–18) of Tat and the SH3 domain (residues 160–212) of Grb2, which impairs activation of the Raf/MAPK pathway and increases the PKA/Raf inhibitory pathway
Histone acetyltransferase KAT2A	NP_066564.2		Binding of HIV-1 Tat to hGCN5 is mediated by amino acids 20–48 of Tat (includes cysteine rich, core, and minimal activation domains of Tat) and by amino acids 111–151 (histone acetyltransferase domain) and 389–476 (bromodomain) of hGCN5
Histone acetyltransferase KAT2B	NP_003875.3		The bromodomain (amino acids 712–832) of P/CAF mediates its binding to amino acids 20–40 of non-acetylated HIV-1 Tat, to amino acids 48–57 in the arginine rich motif of Lys50 acetylated Tat, while Lys28 acetylation of Tat abrogates P/CAF binding to Tat
Histone acetyltransferase KAT5 isoform 1	NP_874369.1		Tip60 is a nuclear histone acetyltransferase that binds to the N-terminal 31 amino acids of HIV-1 Tat
Histone acetyltransferase p300	NP_001420.2		HIV-1 Tat binds to the minimal histone acetyltransferase domain (amino acids 1253–1710) and E1a binding domain (amino acids 1542–1710) of p300, an effect mediated by the basic domain (amino acids 48–57) of Tat
Histone H2A/H2B/H3/H4	NP_003500.1		HIV-1 Tat peptides bind core histones H2A, H2B, H3 and H4, and Tat protein recruits histone acetyltransferases to the HIV-1 LTR promoter leading to acetylation of histones H3 and H4, derepressing chromatin structure and increasing NFkappaB responsiveness
Histone-lysine N-methyltransferase SETD7	NP_085151.1		SET7/9-KMT7 binds directly to HIV-1 Tat and enhances recruitment of the Tat/P-TEFb complex to HIV-1 TAR RNA
Importin subunit alpha-2	NP_002257.1		HIV-1 Tat peptide (amino-acids 47–57) binds to importin alpha and beta receptors
Importin subunit beta-1	NP_002256.2	9891055	The binding of HIV-1 Tat with importin beta is inhibited by RanGTP; HIV-1 Tat peptide (amino-acids 47–57) binds to importin alpha and beta receptors
Insulin-like growth factor-binding protein 4 precursor	NP_001543.2	7778269	Using a yeast two-hybrid system, HIV-1 Tat has been shown to bind the human insulin-like growth factor binding protein 4, suggesting a role for this protein in mediating the biological activity of Tat during HIV-1 replication
Integrin alpha	NP_002196.2		The arginine-glycine-aspartic acid (RGD) sequence present at the carboxy-terminal of HIV-1 Tat mediates vascular cell and monocyte migration and invasion by binding to the alpha-5-beta-1 and alpha-v-beta-3 integrins
Interferon regulatory factor 1	NP_002189.1		HIV-1 Tat represses transcription of the LMP2 gene by competing with STAT1 (signal transducer and activator of transcription 1) for binding to IRF-1 (interferon-regulatory factor-1) at the LMP2 promoter
Interferon-induced, double-stranded RNA-activated protein kinase isoform a	NP_002750.1		Binding of HIV-1 Tat to PKR has been mapped to residues 40–58 of Tat, overlapping the hydrophobic core and basic region of Tat
Lamin isoform A	NP_733821.1		Purified HIV-1 Tat has been shown to bind with high affinity to the nuclear matrix from H9 cells and to link viral RNAs to the nuclear matrix
Lediator of RNA polymerase II transcription subunit 6	NP_005457.2		The interaction of HIV-1 Tat with MED21 hypothetically induces the binding of Tat to MED6
mRNA-capping enzyme	NP_003791.3		HIV-1 Tat binding to mammalian capping enzyme (Mce1) is mediated through the C-terminal domain of Tat (amino acids 49–86) and amino acids 211–597 of Mce1
myoD family inhibitor	NP_005577.1		I-mfa (inhibitor of MyoD family a) and HIC (human I-mfa-domain-containing) proteins serve as substrates for P-TEFb. Their I-mfa domains bind the activation domain of HIV-1 Tat and inhibit Tat- and P-TEFb-dependent HIV-1 transcription
NAD-dependent protein deacetylase sirtuin-1 isoform a	NP_036370.2		HIV-1 Tat binds the deacetylase domain (amino acids 341–512) of SIRT1 and inhibits SIRT1 deacetylase activity, which results in the induction of NF-kappaB hyperacetylation
NF-kappa-B inhibitor alpha	NP_065390.1		Amino acids 72 to 287 of IkappaB-alpha are required for Tat inhibition. Amino acids 263 to 269 within the sixth ankrin of IκB-alpha are required for the binding to Tat
Nuclear factor NF-κB p100 subunit isoform b	NP_002493.3		HIV-1 Tat has been shown to bind NFkappaB in vitro in gel shift, GST-pull down and affinity matrix assays
Nuclear factor of activated T-cells, cytoplasmic 2 isoform B	NP_036472.2		HIV-1 Tat binds to NFAT1, an interaction mediated by the N-terminus of Tat (amino acids 1–26) and the transactivation domain of NFAT1 (amino acids 1–96)
Nuclear inhibitor of protein phosphatase 1 isoform alpha	NP_054829.2		PP1 interacts with Tat in part through the binding of Val (36) and Phe (38) of Tat to PP1, and Tat is involved in the nuclear and subnuclear targeting of PP1
Nuclease-sensitive element-binding protein 1	NP_004550.2		Binding of YB-1 to HIV-1 Tat is mediated through the C-terminal region of Tat (amino acids 48–72) and through amino acids 75–203 of YB-1
nucleophosmin isoform 1	NP_002511.1		The nucleolar shuttle protein B23 binds to HIV-1 Tat and data indicates B23 is necessary for the nucleolar localization of Tat
POU domain, class 2, transcription factor 1 isoform 1	NP_002688.3	7690421	Oct binds to HIV-1 Tat affinity matrices and also confers Tat responsiveness on a basal HIV-1 promoter
Prolow-density lipoprotein receptor-related pr otein 1 precursor	NP_002323.2		LRP binds to the core domain of HIV-1 Tat (amino acids 37–48) and promotes the efficient uptake of Tat into neurons, suggesting Tat may mediate HIV-1 induced neuropathology through disruption of LRP ligands and activation of neuronal genes
Proteasome subunit alpha type	NP_002778.1		HIV-1 Tat binds to the alpha2, alpha4, alpha6, alpha7, beta1, beta2, beta3, beta5, beta6, beta7, LMP7/beta5i, and MECL1/beta2i subunits of the proteasome 20 S core structure and can inhibit cellular proteasome function
Proteoglycan 3 precursor	NP_006084.2		HIV-Tat peptide interferes with polyamine uptake via competition for proteoglycan binding sites rather than a putative downstream transporter in human carcinoma cells
Retinoblastoma-like protein 2	NP_005602.3		HIV-1 Tat protein specifically binds to pRb2/p130 and data suggest this interaction results in the deregulation of the control exerted by pRb2/p130 on the cell cycle, indicating a potential role in AIDS-related oncogenesis
RNA polymerase II subunit A C-terminal domain phosphatase isoform 1	NP_004706.3		FCP1 is required for Tat-mediated transactivation in vitro and amino acids 562–685 of FCP1 are necessary for binding to Tat in yeast two-hybrid studies
Serine/threonine-protein phosphatase PP1-alpha catalytic subunit isoform 1	NP_002699.1		PP1 interacts with Tat in part through the binding of Val (36) and Phe (38) of Tat to PP1, and Tat is involved in the nuclear and subnuclear targeting of PP1
Succinate dehydrogenase [ubiquinone] iron-sulfur subunit, mitochondrial precursor	NP_002991.2	7778269	Using a yeast two-hybrid system, HIV-1 Tat has been shown to bind the human succinate-ubiquinone oxidoreductase iron sulfur subunit, suggesting a role for this protein in mediating the biological activity of Tat during HIV-1 replication
SWI/SNF-related matrix-associated actin-dependent regulator of chromatin subfamily B member 1 isoform a	NP_003064.2		Integrase interactor 1 (INI1)/hSNF5 binds to HIV-1 Tat and co-activates Tat-mediated transcription; both the repeat (Rpt) 1 and Rpt 2 domains of INI1 are required for efficient co-activation
Syndecan-1 precursor	NP_002988.3		Binding of HIV-1 Tat to heparan sulfate proteoglycans is competed out by the heparin-binding factor bFGF; Cell membrane heparin sulfate proteoglycans bind to the basic region of HIV-1 Tat (amino acids 49–57) and act as receptors for extracellular Tat uptake, an effect that may contribute to the angiogenic properties of Tat in promoting Kaposi's sarcoma
TATA-binding protein-associated factor 172	NP_003963.1		HIV-1 Tat binds, through amino acids 36–50, directly to the TBP subunit of the TFIID holoenzyme complex (which includes at least TFIID subunits p250, p125, p70, TBP, and p30), and increases the interaction of TFIID with the HIV-1 LTR promoter
TATA-box-binding protein isoform 1	NP_003185.1		Binding of HIV-1 Tat to TBP has been mapped to the cysteine rich and core domains (amino acids 20–50) of Tat and the H1 alpha helical and S2 domains (amino acids 163–220) of TBP
TFIIH basal transcription factor complex helicase XPB subunit	NP_000113.1		Amino acids 1–48 of HIV-1 Tat, which includes the Tat activation domain, mediate the binding of Tat to CAK and the TFIIH complex through a direct interaction with CDK7 and possibly other TFIIH subunits, including p62 and ERCC3
Thrombospondin-1 precursor	NP_003237.2		Thrombospondin-1 (TSP) binds to HIV-1 Tat, an interaction that can be inhibited by heparin which can bind to both TSP and Tat
Thyroid hormone receptor alpha isoform 2	NP_003241.2	7609079	Thyroid hormone (T3) receptor alpha (T3Ralpha) binds to HIV-1 Tat, an interaction mediated through the DNA-binding domain of T3Ralpha (amino acids 51–118) and the arginine-rich basic region (amino acids 49–57) and possibly amino acids 58–72 of Tat
Transcription activator BRG1 isoform B	NP_003063.2		Acetylated HIV-1 Tat binds efficiently to BRG1 and BAF200 (component of PBAF complex) and weakly to BAF250 (component of BAF complex)
Transcription factor AP-1	NP_002219.1	7690421	Crosslinking experiments suggest a direct binding interaction between HIV-1 Tat and AP1 that is relatively inefficient and that correlates with the ability of AP1 to support Tat transactivation
Transcription factor p65 isoform 1	NP_068810.3		HIV-1 Tat has been shown to bind NFkappaB in vitro in gel shift, GST-pull down and affinity matrix assays
Transcription factor RelB	NP_006500.2		
Transcription factor Sp1 isoform a	NP_612482.2		HIV-1 Tat amino acids 30–55 mediate binding of Tat to Sp1, an effect that some reports indicate is a direct binding interaction, while other reports suggest it is indirect and possibly mediated through interaction with other cellular factors
Transcription initiation factor TFIID subunit 1 isoform 1	NP_004597.2		HIV-1 Tat binds, through amino acids 36–50, directly to the TBP subunit of the TFIID holoenzyme complex (which includes at least TFIID subunits p250, p125, p70, TBP, and p30), and increases the interaction of TFIID with the HIV-1 LTR promoter; Amino acids (aa) 67–101 (C-term. domain) of HIV-1 Tat bind to aa 848–1279 of TAFII250, while Tat aa 18–36 (cysteine-rich domain) and 36–56 (includes basic domain) bind to TAFII250 aa 885–984 (AT domain) and 1120–1279 (Rap74 binding domain), respectively
Transcriptional activator protein Pur-alpha	NP_005850.1		HIV-1 Tat downregulates the expression of p35, a neuron-specific activator of cdk5, and also binds to Puralpha, which associates with cdk5, leading to deregulation of neuronal differentiation and survival
Transportin-1 isoform 1	NP_002261.3	9891055	The binding of HIV-1 Tat with importin beta is inhibited by RanGTP
Tubulin alpha/beta	NP_006000.2		HIV-1 Tat (amino acids 36–39) binds tubulin alpha/beta dimers and polymerized microtubules leading to the alteration of microtubule dynamics and activation of a mitochondria-dependent apoptotic pathway that is facilitated by the Bcl-2 relative Bim
Tumor suppressor protein p73 isoform a	NP_005418.1		Association of tumor protein p73 with HIV-1 Tat prevents the acetylation of Tat on lysine 28 by PCAF, and requires the cysteine-rich domain (amino acids 30 to 40) of Tat, which binds to the N-terminal region (amino acids 1 to 120) of p73
Vascular endothelial growth factor receptor 1 isoform 1 precursor	NP_002010.2	9269752	The mechanism of monocyte activation by HIV-1 Tat involves the binding of Tat to VEGFR-1/Flt-1 and activating signals through this receptor
Zinc finger and BTB domain-containing protein 7A	NP_056982.1		Binding of FBI-1 to HIV-1 Tat is mediated by the zinc finger (ZF) domain of FBI-1 (amino acids 377–584) and is diminished by point mutations in Tat at amino acids 18, 30, and 31

## Competing interests

The authors declare that they have no competing interests.

## Authors’ contributions

AB wrote the manuscript. BES modified parts of the manuscript. Both authors read and approved the final manuscript.
